# The Role of Posturography in the Diagnosis of Temporomandibular Disorders and Their Impact on Body Posture

**DOI:** 10.3390/biomedicines13122857

**Published:** 2025-11-24

**Authors:** Krzysztof Antczak, Waldemar Pluta, Michał Lubkowski, Aleksandra Radecka, Anna Lubkowska

**Affiliations:** 1 Department of Functional Diagnostics and Physical Medicine, Pomeranian Medical University, Zołnierska 54, 71-210 Szczecin, Poland; krzysztof.antczak@pum.edu.pl (K.A.); waldemar.pluta@pum.edu.pl (W.P.); aleksandra.radecka@pum.edu.pl (A.R.); 2 Department of Medical Biology, Pomeranian Medical University, Al. Powstańców Wlkp. 72, 71-210 Szczecin, Poland; michal.lubkowski@pum.edu.pl

**Keywords:** temporomandibular disorders, postural stability, posturography

## Abstract

**Background**: Posturography is a diagnostic method used to evaluate postural stability by recording body sway and the distribution of pressure on the ground. Temporomandibular disorders (TMDs) involve musculoskeletal and neuromuscular dysfunctions affecting the temporomandibular joint, masticatory muscles, and associated structures. Given the anatomical and functional connections between the stomatognathic system and postural control mechanisms, this study aimed to assess whether TMDs influence body posture and balance as measured by posturographic parameters. **Methods**: 75 volunteers, aged 19–48, were included. The TMD group (*n* = 45) was diagnosed based on the Diagnostic Criteria for Temporomandibular Disorders (DC/TMD), and the control group (*n* = 30) showed no signs of TMD. All participants underwent posturographic assessment and jaw opening range measurement. Posturography was performed using a pressure platform that recorded the center of pressure (COP) in static conditions. Postural stability was assessed using the Romberg test with eyes open and closed. **Results**: No statistically significant differences were found between the TMD and control groups in COP parameters, including ellipse area (EA) and total load distribution. Within both groups, COP sway increased significantly in the eyes-closed (EC) condition, as reflected by a greater unsteadiness length (UL). In contrast, EA was larger in the eyes-open (EO) condition in both groups, indicating a wider but more controlled spatial dispersion of COP. Intra-group analysis further revealed a significantly higher load on the left side in the control group only. **Conclusions**: The results do not support a significant postural imbalance in individuals with TMD compared to healthy controls. However, increased sway with eyes closed suggests that visual input plays a key role in postural control, regardless of TMD status.

## 1. Introduction

Posturography is a diagnostic method used to record body movements and assess balance system function. Measurements are obtained by analyzing the displacement of the center of gravity based on foot pressure or through sensors such as accelerometers and gyroscopes [1]. This non-invasive and relatively quick method has proven useful for detecting balance disorders and monitoring rehabilitation progress [2].

Temporomandibular disorders (TMD; ICD-11 DA0E.8) are a heterogeneous group of musculoskeletal and neuromuscular conditions affecting the temporomandibular joint (TMJ), masticatory muscles, and related structures [3]. Their etiology is complex, involving anatomical, biomechanical, and psychosocial factors. Clinical symptoms most often include localized pain, limited mandibular mobility, and joint noises, frequently accompanied by headaches, otologic complaints, or cervical pain [4,5,6].

The diagnostics of TMD have been standardized through validated instruments, such as the Research Diagnostic Criteria (RDC/TMD) and the updated Diagnostic Criteria for TMD (DC/TMD), which allow for reliable classification. There is increasing evidence suggesting a potential link between posture and TMD, although the mechanisms remain unclear [7,8]. Based on this premise, the aim of the present study was to assess the relationship between selected posturographic parameters and the occurrence of TMD. We hypothesized that patients with TMD would exhibit greater instability compared to controls, particularly in the absence of visual input. The primary outcome was defined as unsteadiness length (UL) under eyes-closed conditions, with ellipse area (EA), average sway speed (AS), and load distribution as secondary measures.

## 2. Materials and Methods

This manuscript reports a cross-sectional, observational study designed to assess the relationship between temporomandibular disorders (TMD) and postural stability. This study specifically included patients with myogenous pain-related TMDs with limited mouth opening. Accordingly, the findings should be interpreted within this diagnostic context, and caution is warranted when generalizing the results to other TMD subtypes. The study was conducted and reported in accordance with the Strengthening the Reporting of Observational Studies in Epidemiology (STROBE) guidelines (Supplementary Table S2). The research was conducted from March to June 2024 at the Department of Functional Diagnostics and Physical Medicine of the Pomeranian Medical University in Szczecin. The research was approved by the Bioethics Committee (KB-0012/36/13).

### 2.1. Volunteers

Volunteers for the study were recruited in two ways using an online advertisement. The recruitment process consisted of three stages of qualification, during which individuals with clinical symptoms of TMD and individuals without symptoms were selected. The first stage included the following questions:

1. Have you experienced discomfort related to tension in your temples, jaw, or neck in the last 30 days?
2. Have you experienced pain during chewing, biting, yawning, opening your mouth wide, physical exertion, or under stressful or concentrated conditions in the last 30 days?

Those who answered yes to both questions were initially assigned to the study group (hereinafter referred to as “TMD”). Those who answered no to both questions were initially assigned to the control group. The second stage of qualification included a telephone interview and a short questionnaire to verify the inclusion and exclusion criteria. The inclusion criteria for the study were good health, no orthopedic aids used for mobility, informed consent to participate in the study, age ≥ 18 years, and no contraindications to the study. The exclusion criteria for the study included problems with changing position independently, previous orthopedic treatment, previous splint treatment, use of pharmacotherapy (e.g., oral contraception, hormone replacement therapy, antidepressants), systemic diseases (e.g., rheumatic, metabolic), mental illness, lack of orthopedic stability of the mandible, injuries to the masticatory organ or cervical spine, pregnancy, orthodontic treatment, inflammation in the oral cavity (e.g., pulpitis, impacted molars), smoking, fibromyalgia, and dermatological diseases.

The third stage involved a physical examination, which included palpation of the right and left temporal and masseter muscles and assessment of the maximum range of motion during jaw opening. Among the individuals initially qualified for the TMD group, those with myogenic pain were ultimately included in the study. The physical examination of the control group was aimed at confirming the absence of pain in the examined muscles.

**Figure 1:**
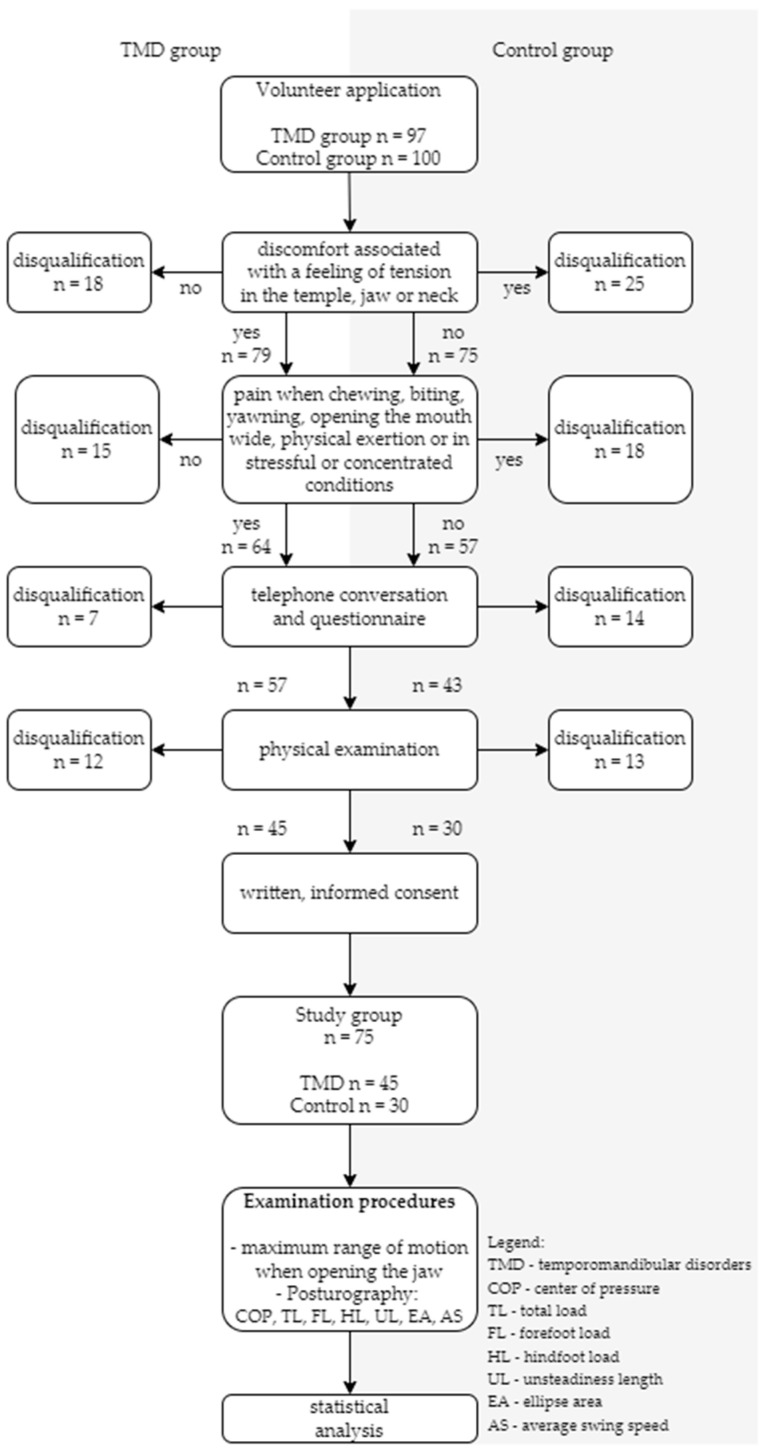
Flowchart of participant recruitment and selection process.

Of the 57 initially qualified individuals, 45 were confirmed in a physical examination to have myofascial pain with limited mouth opening lasting longer than 3 months, in accordance with the DC/TMD criteria (Ib). These individuals were ultimately included in the TMD group. Forty-three individuals initially qualified for the control group underwent additional verification through physical examination. Ultimately, 30 individuals who did not have myofascial pain with limited mouth opening lasting longer than 3 months, according to the DC/TMD criteria (Ib), were included in the control group. After taking into account the inclusion and exclusion criteria and the results of the physical examination, 75 volunteers (including 61 women and 14 men) aged 19 to 48 years ultimately participated in the study. The recruitment process and the sequence of procedures performed are presented in Figure 1.

### 2.2. Research Procedure

#### 2.2.1. Posturography

An advanced FreeMED BASE tensometric platform (40 × 40 cm; Sensor Medica, Guidonia, Italy) was used to analyze postural stability. This device enables precise biomechanical measurements due to tensometric sensors that record the pressure exerted by the feet on the ground. The platform was set to a sampling frequency of 400 Hz, ensuring consistent and accurate acquisition of COP data.

The study was conducted under controlled laboratory conditions (ambient temperature 21–22 °C; complete silence) to minimize external influences. Patients stood barefoot on the platform with a standardized stance width of shoulder distance and feet oriented at an angle of approximately 15°. The arms hung freely along the torso, and the gaze was fixed on a visual target positioned at eye level, 2 m in front of the participant. The mandibular position remained habitual during testing.

Each participant performed the Romberg test in two conditions (eyes open, eyes closed). The order of conditions was randomized, and a 1-min rest interval was provided between trials to minimize fatigue. Each recording lasted 51.2 s.

The platform recorded the center of pressure (COP), analyzed as both a static parameter (mean COP position) and a dynamic parameter (COP displacement over time) in the sagittal and frontal planes. The following stabilometric indicators were calculated: Total Load (TL), Forefoot Load (FL), Hindfoot Load (HL), Unsteadiness Length (UL), Ellipse Area (EA), and Average Swing Speed (AS).

#### 2.2.2. Limitation Concerning Reliability

Although FreeMED/FreeStep systems have previously demonstrated good-to-excellent test–retest reliability for static stabilometric measures (intraclass correlation coefficients [ICC] typically 0.75–0.95) in both healthy individuals and patients with balance impairments [9] we did not conduct a formal reliability substudy within our cohort. This may limit the internal consistency assessment of the current results. We therefore acknowledge that the absence of in-house reliability testing represents a methodological limitation and may restrict the external generalizability of our findings.

A limitation of the present study is the absence of published ICC values for COP parameters measured with the FreeMED/FreeStep system under conditions identical to ours (Romberg stance, 51.2-s trials, EO/EC). Although moderate to good reliability has been reported for comparable COP metrics in similar tasks [10] these findings cannot be directly extrapolated to our protocol. Future research should include a dedicated test–retest substudy to establish ICCs specific to this platform and measurement setting.

### 2.3. Statistical Analysis

Statistical analysis was performed using Statistica 13.3 software (Statistica PL, StatSoft, Krakow, Poland), assuming a significance level of *p* < 0.05. Data distribution was assessed using the Shapiro–Wilk test and a scatter plot. For normally distributed data, the characteristics of the variables were presented as arithmetic means and standard deviations. For variables whose distribution deviated from normal, the median with interquartile range values were used. Student’s *t*-test or Mann–Whitney U test was used to assess the significance of differences between pairs of variables, depending on the distribution of the data. The results obtained from the stabilometric platform were analyzed using multivariate analysis of variance (MANOVA). Given the higher proportion of females in the TMD group compared to controls, multivariate analysis of covariance (MANCOVA) was additionally performed with adjustment for potential demographic confounders including sex, height, and BMI to control for their potential influence on center of pressure (COP) metrics. Due to the non-normality of the distribution for parameters such as ellipse area and swing length, Box–Cox transformations were used to normalize the data. Height and BMI variables were centered (mean-centered) before inclusion as covariates in the MANCOVA model to facilitate interpretation and reduce multicollinearity. Intra-group analyses were performed using contrast analysis. Multiple planned contrasts following MANOVA (EO vs. EC, left vs. right, TMD vs. control) were based on theoretically motivated a priori hypotheses and did not require family-wise error rate correction.

Post-hoc power analysis was conducted to determine minimal detectable effect sizes at the given sample sizes (*n*_1_ = 30 TMD, *n*_2_ = 45 controls) with α = 0.05 and power = 0.80. For normally distributed data, Cohen’s d was calculated along with 95% confidence intervals. For non-parametric variables, Cliff’s delta (δ) was computed using bootstrap resampling (1000 iterations) to estimate confidence intervals. Detailed effect size analysis is presented in Supplementary Table S1.

## 3. Results

The completed STROBE checklist is included below, demonstrating compliance with the guidelines for reporting cross-sectional studies (Table 1).

### 3.1. Characteristics of the Study Group

The study group consisted of 75 volunteers (61 women, 14 men). The median age of the participants was 27.0 years (range: 19–48). There were no significant differences in age between the TMD group (median: 26.0 years, range: 19–48) and the control group (median: 28.5 years, range: 19–46). Similarly, no significant differences were found between the TMD and control groups in the assessed anthropometric parameters, such as body weight (median: 68.00 kg vs. 72.00 kg), body height (median: 1.69 m vs. 1.71 m), and BMI (median: 23.44 kg/m^2^ vs. 25.26 kg/m^2^). The clinical parameter of maximum mouth opening (MMO) was also analyzed. In the TMD group, the mean MMO was 3.98 cm (±0.752), while in the control group, it was significantly higher, amounting to 4.48 cm (±0.696) (*p* < 0.01). Furthermore, the analysis of posturographic parameters did not reveal any statistically significant differences between the TMD and control groups. This applied to load distribution, including the mean total load on the left foot (TMD: 50.11% vs. Control: 51.21%) and right foot (TMD: 49.89% vs. Control: 48.79%), as well as forefoot and hindfoot load percentages. Likewise, no significant inter-group differences were observed in parameters measuring postural sway, such as unsteadiness length (UL), ellipse area (EA), and average swing speed (AS), under both eyes-open (EO) and eyes-closed (EC) conditions. The characteristics of the study group are summarized in Table 2.

Power analysis revealed that with our sample sizes, the minimal detectable Cohen’s d was 0.669 for parametric variables and minimal detectable Cliff’s delta was approximately 0.364 for non-parametric measures. Most observed effect sizes fell below these detection thresholds, explaining the non-significant group differences (Supplementary Table S1).

### 3.2. Analysis of the Ellipse Area and Deflection Lengths

Multivariate analysis of covariance (MANCOVA) with adjustment for sex, height, and BMI was performed to control for potential demographic confounders, given the higher proportion of females in the TMD group.

#### 3.2.1. Ellipse Area Analysis

**Figure 2:**
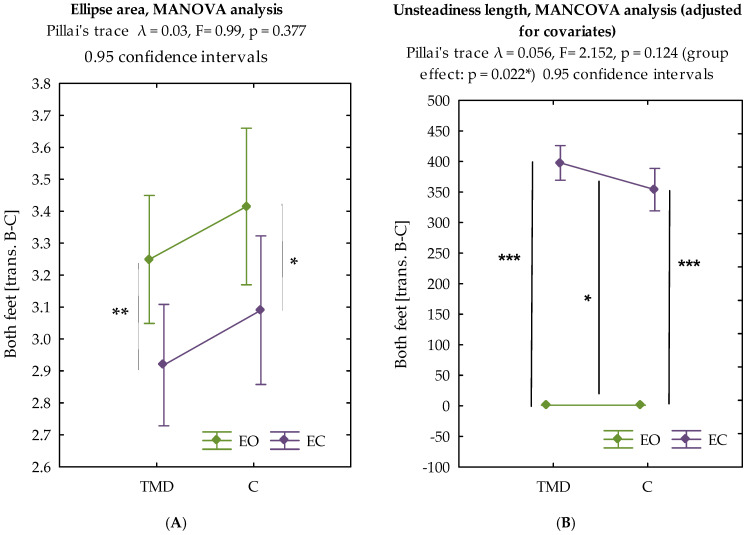
Interaction plot for transformed data of ellipse area and unsteadiness length for study groups. (**A**) Ellipse area [transformed values] for eyes open (EO) vs. eyes closed (EC) conditions; (**B**) Unsteadiness length [transformed values] for EO vs. EC conditions. Legend: TMD = temporomandibular disorders; C = control group; EO = eyes open; EC = eyes closed. Legend: *—*p* < 0.05; **—*p* < 0.01; ***—*p* < 0.001, between groups (adjusted for sex, height, BMI).

MANCOVA did not reveal statistically significant differences between the TMD and control groups in the values of the transformed ellipse area data (Pillai’s trace λ = 0.027, F = 0.990, *p* = 0.377). The covariate adjustments (sex: *p* = 0.411; height: *p* = 0.816; BMI: *p* = 0.051) did not alter the primary finding. In both the TMD group (*p* < 0.01) and the control group (*p* < 0.05), the ellipse area value was significantly greater in the open-eye test compared to the closed-eye test (Figure 2A).

#### 3.2.2. Unsteadiness Length Analysis

MANCOVA revealed statistically significant differences between the groups in the values of the transformed unsteadiness length data after covariate adjustment (Pillai’s trace λ = 0.056, F = 2.152, *p* = 0.124 for the overall multivariate test; group effect: *p* = 0.022). This finding emerged only after controlling for demographic variables, highlighting the importance of covariate adjustment in posturographic analyses. BMI showed a borderline significant effect (*p* = 0.051), while sex (*p* = 0.411) and height (*p* = 0.816) did not significantly influence COP metrics. In both groups of subjects, the value of deflection length was greater in the eyes-closed test (*p* < 0.001) (Figure 2B).

### 3.3. Analysis of Variance

**Figure 3:**
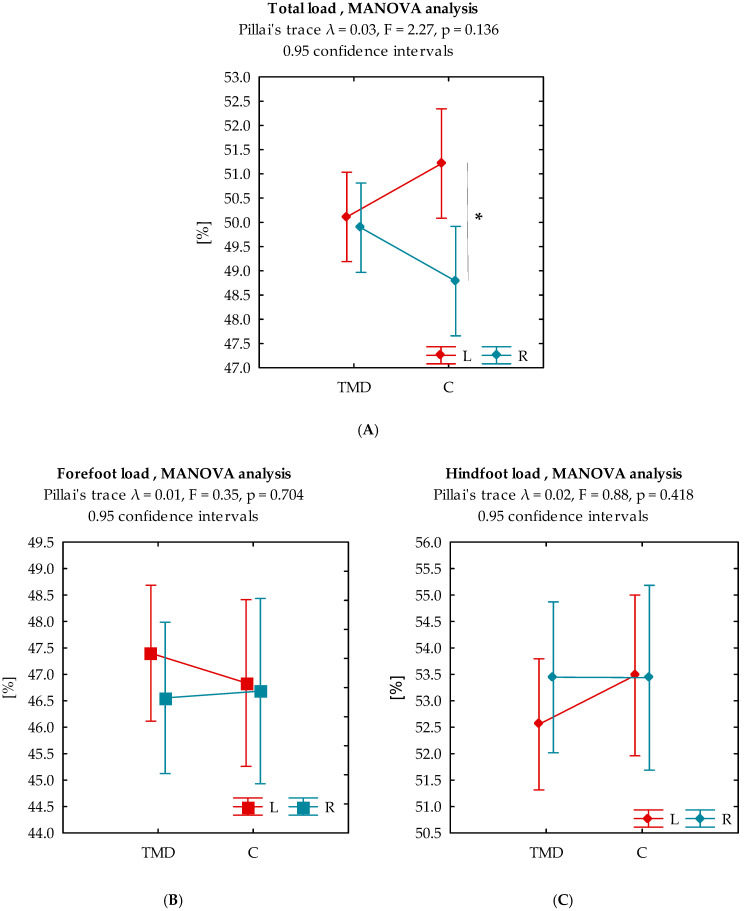
Interaction plot for total, forefoot and hindfoot load for study groups. (**A**) Total load distribution between left (L) and right (R) feet; (**B**) Forefoot load distribution [%]; (**C**) Hindfoot load distribution [%]. Legend: TMD = temporomandibular disorders; C = control group; L = left foot; R = right foot; *—*p* < 0.05.

Multivariate analysis of variance did not reveal any statistically significant differences between the TMD group and the control group (Pillai trace λ = 0.030, *p* = 0.136) in terms of total load values. Detailed intra-group analysis showed statistically significant (*p* < 0.05) higher total load values on the left side only in the control group (Figure 3A).

The analysis of forefoot and hindfoot load did not reveal any statistically significant differences between groups (Pillai trace λ = 0.010, *p* = 0.704 for forefoot and Pillai trace λ = 0.024, *p* = 0.418 for hindfoot) or within groups (Figure 3B,C). Table 3 summarizes the results of the contrast analysis of the variables discussed. The corresponding contrast analysis outcomes for these parameters are also shown in Table 4.

## 4. Discussion

Our results provide further evidence that postural control mechanisms may be altered in patients with temporomandibular disorders (TMD). Several biomechanical and neuromuscular pathways may underlie this association. Abnormal postural habits, such as reduced cervical curvature or increased cranio-cervical angles, have been suggested as predisposing factors [9,10]. Moreover, the biomechanics of the feet play a fundamental role in maintaining overall stability, and dysfunctions at this level may induce compensatory changes in the pelvis, spine, and cranio-cervical region, ultimately influencing mandibular biomechanics [11,12]. In addition, patients with TMD have been shown to exhibit increased muscle tension at rest and reduced efficiency during clenching, which may be linked to postural or biomechanical dysfunctions [13]. Although direct causal relationships remain unconfirmed, these findings support the concept of multidirectional interactions between posture control and temporomandibular joint function [14,15].

The balance analysis in the posturography study did not reveal any statistically significant differences between the two groups in the presented studies (Table 2 and Table 3). The literature presents varying findings regarding the relationship between TMD and cervical spine alignment, with some studies indicating a correlation between TMD and changes in cervical curvature and POCA [12,16]. This phenomenon can potentially induce biomechanical alterations not only in the cervical segment, but also in the lower parts of the spine, which is reflected in the parameters quantified during posturographic examination [13]. For this reason, an important element of our study was to assess the impact of TMD on body posture and balance test results in posturographic testing. However, no differences were found between the group with diagnosed TMD and healthy volunteers in the values of total foot load indices. Techniques such as three-dimensional scanning and raster stereography, which enable a full-body scan of the patient, allow for a more precise analysis of the relationship between TMD dysfunction and body posture. In a 2024 study, Hampe et al. performed a three-dimensional scan of the entire body in a standing and sitting position using a Vitus Smart XXL 3D scanner (Human Solutions, Kaiserslautern, Germany) [17]. The measurement was performed twice, before and after the application of occlusal splints in patients with diagnosed TMD symptoms (RC/TMD). The results clearly indicated postural changes in the patients’ bodies, but these changes only affected the upper body [14]. Di Paolo et al. came to similar conclusions when they used rasterstegography, a non-invasive, radiation-free method of three-dimensional analysis of the spine, to assess the posture of patients with TMD. The researchers demonstrated the influence of TMD on lateral and rotational deviations of the upper spine [18]. More sensitive methods provide a deeper understanding of the complex relationship between temporomandibular joint dysfunction (TMD) and body posture, which may lead to more effective treatment strategies.

There are increasing reports in the literature on the direct impact of TMD on myofascial dysfunction in relation to the patient’s entire body and postural habits. However, only a very limited number of studies have specifically investigated this relationship [19,20]. These results may be influenced by the characteristics of the static posturography test itself. This method does not fully capture myofascial interactions or postural adaptation mechanisms, which are important in everyday, variable functional conditions. As a result, static measurements may underestimate the complexity of postural regulation mechanisms, affecting the interpretation of the relationship between TMD and myofascial dysfunctions.

Studies on static and dynamic posturography protocols have shown that body balance reference values vary depending on age group and gender. Based on their own research, Nishino and colleagues identified three age groups: 20–59 years, 60–69 years, and 70–89 years for women and men. Older people obtain higher scores in posturographic tests, which results in higher standards being adopted for this group [21]. All patients participating in our study belong to the first age group defined by the above authors.

Our study did not reveal any significant differences in body posture between individuals with temporomandibular disorders (TMD) and those without disorders. The posturography method using a mat mainly records changes in the area of foot contact with the ground, without providing direct data on the biomechanics of the upper body segments. However, interestingly, our analysis revealed distinct postural control strategies between TMD patients and controls. TMD patients demonstrated larger ellipse areas despite shorter path lengths compared to controls, suggesting different biomechanical approaches to postural regulation. This pattern indicates that TMD patients may employ a more rigid postural control strategy, occupying larger sway envelopes while making fewer corrective movements. This phenomenon could reflect altered sensorimotor integration or compromised proprioceptive feedback associated with TMD, leading to delayed postural corrections and muscle co-contraction patterns that create postural rigidity. Importantly, after adjusting for demographic variables (sex, height, BMI) using multivariate analysis of covariance (MANCOVA), a statistically significant difference in unsteadiness length was revealed between TMD patients and controls (group effect: *p* = 0.022). This emphasizes the necessity of covariate adjustment in posturographic studies, as group differences may remain undetected or be confounded without appropriate statistical control (Figure 2B).

The interpretation of posturographic results in the context of temporomandibular disorders should take into account the fact that posturography primarily assesses the end result of complex posture control processes, rather than the mechanisms of these processes directly. In addition, compensatory mechanisms of the central nervous system may mask the impact of temporomandibular joint dysfunction on overall postural stability [22].

This study has several limitations that should be acknowledged. Firstly, the primary methodological limitation is the use of traditional static posturography, which may not be sensitive enough to detect subtle postural changes, particularly asymmetries in the upper body that might be associated with TMD. Additionally, compensatory mechanisms of the central nervous system could mask the actual impact of TMD on overall postural stability during a static test.

Secondly, the generalizability of the findings may be limited by the characteristics of the study sample. The relatively small sample size (*n* = 75) and the inclusion of a specific subgroup of patients—those with myogenic, myofascial pain—mean that the results may not apply to all types of temporomandibular disorders.

Finally, the cross-sectional design of the study allows for the identification of associations but does not permit the establishment of a cause-and-effect relationship between TMD and postural imbalance. The primary aim of the study was to explore these associations and describe potential links between the variables rather than to demonstrate causality. The study title and objectives were formulated to reflect this aim, and this limitation has been clarified in the manuscript to ensure transparency regarding the scope of our findings.

## 5. Conclusions

The effectiveness of posturography as a method for assessing the co-occurrence of balance disorders with TMD is limited. Traditional posturography does not account for subtle postural changes, such as asymmetries in the upper body, which may be associated with TMD. Modern techniques, such as 3D scanning and rasterstegography, offer more precise tools for analyzing these relationships.

Although TMD can affect balance control mechanisms by modulating neck muscle tension, conventional posturographic methods do not provide sufficient data for a comprehensive evaluation of these interactions. Research findings suggest that the impact of TMD on balance is subtle and difficult to detect using standard posturography protocols.

## Figures and Tables

**Table 1:** STROBE checklist.

	Item No.	Page No.
Recommendation		
Title and abstract	1	1
Introduction		
Background/rationale	2	1–2
Objectives	3	2
Methods		
Study design	4	3
Setting	5	3–5
Participants	6	3–5
Variables	7	5–9
Data sources/measurement	8	3–5
Bias	9	4–5
Study size	10	5
Quantitative variables	11	5
Statistical methods	12	5
Results		
Participants	13	3–5
Descriptive data	14	3–6
Outcome data	15	5–9
Main results	16	5–9 Supplementary material Table S1
Other analyses	17	5–10
Discussion		
Key results	18	11
Limitations	19	12
Interpretation	20	12
Generalisability	21	11–12
Other information		
Funding	22	14

**Table 2:** Characteristics of the study group.

	Study Group *n* = 75 ♀ = 61, ♂ = 14	TMD *n* = 45 ♀ = 38, ♂ = 7	Control Group *n* = 30 ♀ = 23, ♂ = 7	TMD vs. C
	Mean ± SD/IQR	Mean ± SD/IQR	Mean ± SD/IQR	U-M-W/Test-T
age [years]	27.00 [22.00–33.50]	28.50 [23.00–34.75]	26.00 [22.00–32.00]	−0.50
weight [kg]	71.00 [60.00–80.00]	72.00 [68.25–80.00]	68.00 [59.00–79.00]	−1.41
height [m]	1.69 [1.64–1.75]	1.71 [1.65–1.80]	1.69 [1.64–1.72]	−1.20
BMI [kg/m^2^]	24.69 [21.26–26.84]	25.26 [22.72–26.64]	23.44 [21.01–27.25]	−0.96
MMO [cm]	4.18 ± 0.76	3.98 ± 0.75	4.48 ± 0.70	−2.85 **
TL LF [%]	50.55 ± 3.13	50.11 ± 2.63	51.21 ± 3.71	−1.51
TL RF [%]	49.45 ± 3.13	49.89 ± 2.63	48.79 ± 3.71	1.51
FL LF [%]	47.18 ± 4.31	47.40 ± 4.08	46.84 ± 4.68	0.55
FL RF [%]	46.61 ± 4.78	46.56 ± 4.20	46.68 ± 5.62	−0.11
HL LF [%]	52.93 ± 4.17	52.56 ± 4.05	53.48 ± 4.36	−0.94
HL RF [%]	53.44 ± 4.77	53.44 ± 4.20	53.44 ± 5.59	0.01
UL EO [mm]	336.36 [304.39–387.56]	328.36 [291.61–368.61]	344.07 [317.62–404.14]	1.47
UL EC [mm]	363.40 [308.38–427.41]	330.86 [287.72–385.44]	379.33 [315.40–440.63]	1.55
EA EO [mm^2^]	54.82 [30.91–115.53]	61.25 [41.80–145.92]	47.66 [27.40–90.25]	−1.30
EA EC [mm^2^]	35.12 [17.64–57.19]	38.91 [28.17–47.25]	24.70 [15.78–59.50]	−1.09
AS EO [mm/s]	7.12 [6.54–8.28]	6.95 [6.11–7.99]	7.30 [6.70–8.65]	1.53
AS EC [mm/s]	7.77 [6.62–8.87]	7.72 [6.64–8.77]	7.85 [6.62–8.87]	0.21

Legend: Data are expressed as mean with standard deviation (SD) or median with interquartile range values (IQR). BMI—body mass index; MMO—maximum mouth opening, TL—total load; FL—forefoot load; HL—hindfoot load; LF—left foot; RF—right foot; UL—unsteadiness length; EA—ellipse area; AS—average swing speed; EO—eyes open; EC—eyes closed; ♀—women; ♂—men, ** *p* < 0.05.

**Table 3:** Contrast analysis results for ellipse area and unsteadiness length for study groups.

Ellipse Area				
	TMD		C	
	EO	EC	EO	EC
EO	x	−2.658 **	x	−2.129 *
EC	x	x	x	x
Unsteadiness length				
	EO	EC	EO	EC
EO	x	−27.947 ***	x	−20.302 ***
EC	x	x	x	x

Legend: *—*p* < 0.05; **—*p* < 0.01; ***—*p* < 0.001.

**Table 4:** Contrast analysis results for total, forefoot and hindfoot load for study groups.

Total Load				
	TMD		C	
	L	R	L	R
L	x	−0.240	x	−2.141 *
R	x	x	x	x
Forefoot load				
	L	R	L	R
L	x	−1.504	x	−0.224
R	x	x	x	x
Hindfoot load				
	L	R	L	R
L	x	1.718	x	−0.069
R	x	x	x	x

Legend: *—*p* < 0.05.

## Data Availability

Data are contained within the article. Raw data for individual measurements available on request from the authors.

## Supplementary Materials

The following supporting information can be downloaded at: https://www.mdpi.com/article/10.3390/biomedicines13122857/s1, Table S1: Power analysis and effect sizes for group contrasts between TMD patients and healthy controls; Table S2: STROBE checklist for cross-sectional studies.
